# Risk Awareness and Attitude of German Farmers towards Biosecurity Measures

**DOI:** 10.3390/ani14071102

**Published:** 2024-04-04

**Authors:** Anna Herrmann, Katja Schulz, Natalie Wischnewski, Jule Brüssau, Eva Zeiler, Carola Sauter-Louis

**Affiliations:** 1 Institute of Epidemiology, Friedrich-Loeffler-Institut, Südufer 10, 17493 Greifswald-Insel Riems, Germany; anna.herrmann@fli.de (A.H.); katja.schulz@fli.de (K.S.); j.bruessau@web.de (J.B.); carola.sauter-louis@fli.de (C.S.-L.); 2 Faculty of Sustainable Agricultural and Energy Systems, University of Applied Sciences Weihenstephan-Triesdorf, Am Staudengarten 1, 85354 Freising, Germany; eva.zeiler@hswt.de

**Keywords:** biosecurity, farm management, animal husbandry, farmer’s attitude, online-survey, Germany

## Abstract

**Simple Summary:**

Biosecurity measures are crucial for protecting farms against the introduction of animal diseases, given the dynamic nature of outbreaks. Livestock farmers play a key role in the success of these strategies. To understand German farmers’ perspectives on biosecurity, an anonymous online survey was conducted. Alongside general farm information, data were collected on existing biosecurity protocols and potential disease entry pathways, along with corresponding protective measures. The evaluation of the survey results indicates farmers’ awareness of the importance of biosecurity, evidenced by the presence of biosecurity concepts on their farms. While farmers recognize potential disease entry points, there is a lack of knowledge on improving biosecurity in these areas. Overall, farmers’ high commitment to biosecurity suggests routine implementation of measures. However, their lack of knowledge about the effectiveness of biosecurity measures needs to be further investigated in future studies.

**Abstract:**

The implementation of management activities depends on both the attitude of the people performing the work and their understanding of why the work should be performed. In the context of animal husbandry, the implementation of such practices is crucial for the functionality of biosecurity. Therefore, it is important to know how farmers perceive biosecurity as a whole. An anonymous online survey was conducted among German farmers. In addition to general data about their farm, information about their existing concept of biosecurity, as well as about the assessment of possible introduction routes for animal diseases into the farm with regard to their likelihood, was gathered. Furthermore, information on measures to protect their farm against disease introduction were retrieved. Analysis showed that in general, farmers were aware of the importance of biosecurity and consequently had concepts of biosecurity on their farms. However, awareness about dangerous introduction routes for animal diseases into a farm was associated with a lack of knowledge of how to improve the measures in these areas. The role of the veterinarian in the context of biosecurity was highlighted and further problematic areas were indicated. Overall, the high level of commitment from farmers indicated a good implementation of daily practices.

## 1. Introduction

Pathogens infecting animals kept for food production pose a threat on multiple layers. Not only is the health and therefore the wellbeing of the livestock in threat [1,2], but also the health of the humans consuming the produced food [3,4,5]. Furthermore, losses of animals along the production chain reduce the efficient use of resources, leading to an economic loss as well as a higher impact on the environment per produced unit [6]. The concept of biosecurity generally deals with the elimination or at least the minimization of biological contamination [7]. With regard to animal health and production systems, the term biosecurity is defined by the World Organization for Animal Health (WOAH) as “a set of management and physical measures designed to reduce the risk of introduction, establishment and spread of animal diseases, infections or infestations to, from and within an animal population” [8]. Other definitions focus more on the link of animal diseases to public health and thereby highlight the importance of the One Health concept [9]. Biosecurity on a farm can be divided into external biosecurity, ensuring that no pathogens enter the farm, and internal biosecurity, hindering the spread of pathogens within the farm [10]. In addition, a distinction can be made between operational biosecurity, which describes management practices, and constructional biosecurity, which includes building measures [11]. While constructional biosecurity can be controlled by national authorities, the actual daily implementation of management practices on the farm cannot. Therefore, the success of biosecurity measures is strongly dependent on their implementation on site. Thus, the people in charge of the animal facility represent the key figures in a successful biosecurity concept [12]. Certain socio-psychological factors affect the choice of a farmer of whether to implement biosecurity measures on their farm or not [13,14]. The awareness of the problem as well as the perceived effectiveness and feasibility of a measurement are thereby the main factors of the probability of its adaption [15,16].

In Germany, animal production systems are currently threatened by the circulation of highly pathogenic avian influenza (HPAI) [17] as well as African swine fever (ASF) [18]. Therefore, functioning biosecurity on a farm level is crucial in order to prevent outbreaks. Evaluating the current situation regarding biosecurity measures in Germany, an online survey was conducted among farmers. The aim was to assess the risk awareness in regards to pathogen introduction to the farm, the self-perception of the farm’s biosecurity status and the general attitude of farmers towards biosecurity. The collected data allow for an estimation about the functionality of the operational biosecurity on farms in Germany.

Even though biosecurity measures are implemented to hinder the introduction and spread of all kinds of diseases into and within the farm, some infectious animal diseases like HPAI and ASF are listed by the European Union (EU) law (Regulation (EU) 2016/429 (Animal Health Law)) and are associated with a specific series of control measures. The term ‘Tierseuchen’ used in the original German questionnaire was translated as ‘animal diseases’ for the writing of the present paper, but implies that the animal disease is listed by the EU. The focus of the presented paper lies therefore on these listed animal diseases.

## 2. Materials and Methods

### 2.1. Recruitment of Participants

An anonymous online survey was conducted among farmers that keep farm animals for the production of food. More precisely, people that owned a farm or were hired to oversee an animal facility were encouraged to participate. All German farmers and agricultural workers who were involved in biosecurity issues and reachable through the selected distribution system were encouraged to participate in the survey. There was no selection based on regions or production types. All completed datasets were considered in the survey analysis. Due to significant differences in husbandry and management practices, fish and bee holdings were not included in this questionnaire. The survey was accessible starting from the 1st of March and until the 31st of May 2023, leading to a conscience sampled data pool [19]. The recruitment of participants was achieved through indirect snowball sampling [20,21]. In snowball sampling, participants or survey respondents were requested to invite additional individuals who belonged to the target group to participate in the survey. Altogether, three social media influencers that focused on agricultural topics with a focus on political themes, three governmental institutions, three industrial companies, fourteen media outlets in the field of agricultural news and thirty farmers’ associations were addressed in the beginning of March 2023 simultaneously with the start of the survey. Nine addressees responded, of which eight released a call for participation on various social media platforms or sent an informational letter to their members. Further announcements were posted on various platforms of the contacted institutions, even though they did not respond to the authors’ initial request. The organizations contacted were only involved in the distribution of the survey. Nevertheless, it was possible for individual employees of the organizations or agricultural influencers who, for example, managed a farm and were confronted with the issue of biosecurity in their day-to-day operations to take part in the survey.

### 2.2. Questionnaire

The survey was designed using the ScoSci Survey software (version 3.4.10; https://www.soscisurvey.de; [22] accessed on 13 July 2023) and was made available via this website. It consisted of ten questions about the structure of the farm, the state of its biosecurity and the awareness of potential threats as well as potential measures of improvement. Pretesting was performed by seven veterinarians working in the field of epidemiology and three agronomists working in the field of animal husbandry. The questionnaire can be found in Supplementary S1.

The questions were presented on four pages headlined and categorized by topics. First, general information was collected via four questions. Information about the respondent’s number of years of experience in the husbandry of farm animals, the type of production system, the type of animal species kept on the farm and the location of the farm on a federal state level was collected. The question about the species kept on the farm had respective subcategories concerning the type of production or the species. For each subcategory, except for the subcategories of the ‘Ungulates’, the number of animals kept in that category was asked, using three categories of ranges.

The second category was labelled state of biosecurity. It contained a question with five statements about biosecurity on their farm that participants were asked to evaluate according to a five-point Likert scale [23]. Statements are abbreviated in the following text as described in Table 1.

Subsequently, the participants were asked from which source they obtained the information for the development and maintenance of the biosecurity concept on their farm. Furthermore, they were asked about the revision of their biosecurity plan and the tools used in that context.

In the third category, a picture with twelve pictograms combined with respective subtitles of twelve introduction routes for pathogens into the farm was displayed. Two statements were given, accompanied by a distinctive symbol: ‘Here I see a high risk of disease introduction into my farm!’ and ‘Here my farm is very well positioned in terms of biosecurity!’. Participants were asked to allocate each statement to, at most, three of the displayed introduction routes by clicking into the respective pictogram (Supplementary S1).

In the last category, one question was asked about possible offers that would interest the participants for improved biosecurity on their farm. The opt-out option was that no offers were needed. Finally, an open text field question was displayed for further thoughts on the topic of on-farm biosecurity.

### 2.3. Data Inclusion

In order to raise the participation rate, answering was made optional for all questions. All interviews that were submitted by pressing the button “send data” at the last page of the survey were considered as datasets. For the final analysis, datasets that did not include any answers and datasets that indicated that the farm was located in Austria or in Switzerland were excluded, as well as farms that indicated that they kept fish or bees only.

### 2.4. Data Analysis

Data were analyzed descriptively as well as exploratively. For descriptions of farm sizes, the number of animals per animal species kept was analyzed and categorized. The median was calculated with the mean absolute deviation (MAD). Farms that kept at least one species subgroup of the highest given number category were categorized as large. Every other farm that had information about at least one animal species kept was categorized as small. In parts, animal species other than cattle, swine and chicken were summarized under the term ‘other animal species’ for a more comprehensive description. Similarly, the answers ‘Does rather apply’ and ‘Does apply’ were summarized as ‘agreed’ for parts of the interpretation.

The correlation between the location of the participating farms and the total number of farms keeping animals in Germany was analyzed by a simple linear regression model. In addition, a correlation analysis was performed with a subset of data for each animal species kept. The data concerning the total farms registered in Germany were obtained from the Federal Statistical Office of Germany. For the overall number of farms as well as the number of farms keeping chicken, goats and poultry, data from March 2020 were used [24]. For the number of registered farms keeping cattle, pigs and sheep, more recent data from November 2022 were available [25]. The correlation of answers given to the statements about attitudes towards biosecurity were analyzed using Spearman’s rank correlation coefficient (ρ) [26] and interpreted according to Cohen, whereby correlation coefficients larger than 0.5 were considered as strong correlations [27]. The *p*-values for Spearman correlation were calculated using asymptotic t approximation with Edgeworth series approximation. Analysis included all datasets that dealt with all five statements. In all correlation analyses, a *p*-value of less than 0.05 was considered significant. Thematic text analysis was performed on the answers from the open question, coding the information given in the text answer according to Braun and Clarke [28]. Therefore, after familiarization with the data, codes were generated according to features found in the data. These codes were then combined into overarching themes. Statistical analysis was performed in R [29] using the package psych [30]. For the creation of figures, the packages ggplot2 [31], pheatmap [32], corrplot [33] and UpSetR [34,35] were used.

## 3. Results

### 3.1. Properties of Participating Farms

**Figure 1:**
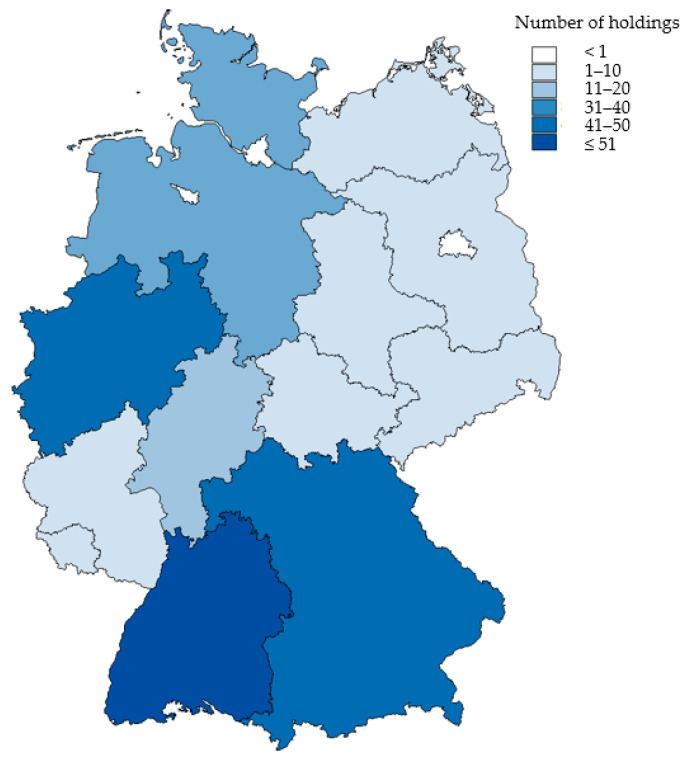
Number of farms per federal state. Four Austrian and two Swiss holdings also took part. Five holdings did not provide any information on their situation.

Overall, 239 complete datasets were acquired. Farms were described to be located in 13 out of the 16 German federal states, with most farms located in Baden-Württemberg (*n* = 51, 21.3%), North Rhine-Westphalia (*n* = 45, 18.8%) and Bavaria (*n* = 41, 17.2%). None of the farms were located in the three city-states (Berlin, Hamburg, Bremen) (Figure 1).

**Figure 2:**
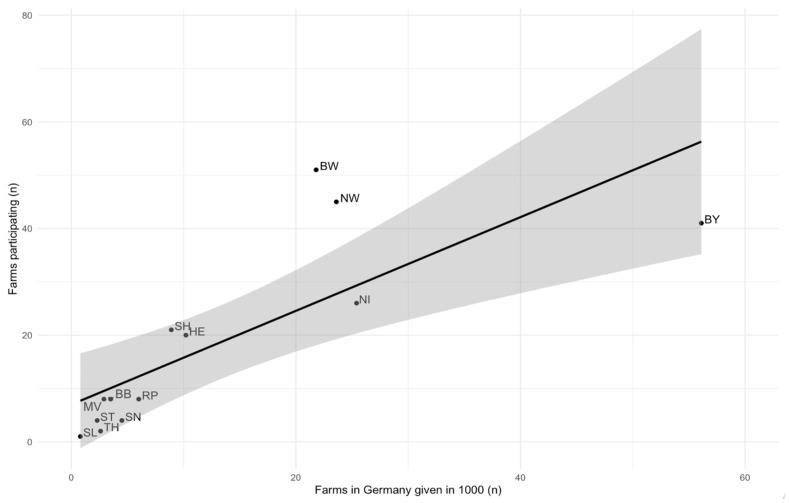
Univariable linear regression model of participating farms from each German federal state in dependence of farms registered in Germany. Line fits represent linear regression and 95% confidence interval. BB = Brandenburg, BW = Baden-Württemberg, BY = Bavaria, HE = Hesse, MV = Mecklenburg-Western Pomerania, NI = Lower Saxony, NW = North Rhine-Westphalia, RP = Rhineland-Palatinate, SH = Schleswig-Holstein, SL = Saarland, SN = Saxony, ST = Saxony-Anhalt, TH = Thuringia.

The proportion of farms participating in the study complied (*p* = 0.00, R2 = 0.58) with the proportion of farms in Germany (Figure 2). Furthermore, the proportion of participating farms keeping cattle (*p* = 0.02, R2 = 0.35), pigs (*p* = 0.00, R2 = 0.79) and poultry (*p* = 0.05, R2 = 0.24) complied with the proportion of registered German farms keeping the respective animal species. In contrast, farms that stated that they kept sheep (*p* = 0.14, R2 = 0.11), other poultry (*p* = 0.78, R2 = −0.08), ungulates (*p* = 0.23, R2 = 0.05) and goats (*p* = 0.12, R2 = 0.13) did not comply with the proportion of these farms in Germany.

**Figure 3:**
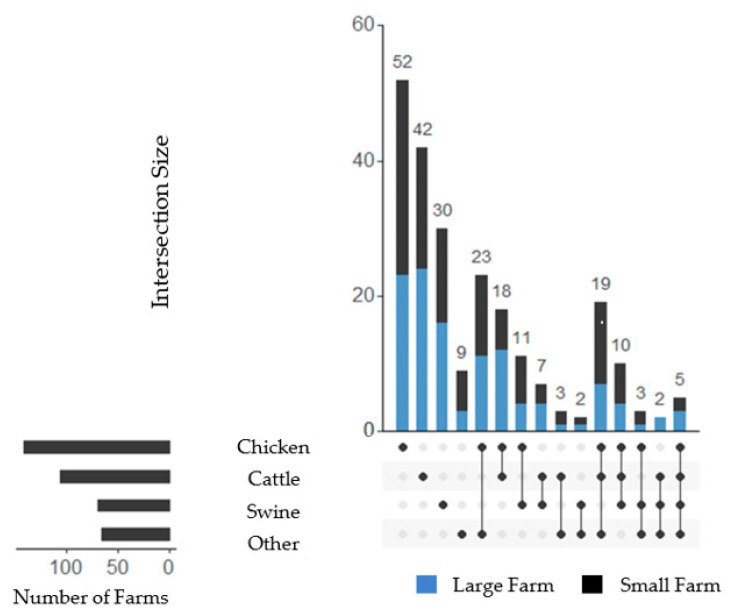
Animal species kept on the farms of participants. The number of farms keeping the respective animal species is given on the vertical columns on the right side. The vertical columns give the number of farms that kept animals of a specific species or resp. multiple species, as indicated underneath the bars by the black dot(s) in combination with the black lines in between them, indicating a combination of these species. The number of farms categorized as large in each category is highlighted in blue. Classification for cattle and other animals: S = 1–99 animals, L = more than 100 animals; for swine and chicken: S = 1–999 animals, L = more than 1000 animals.

Participants stated in most cases that they had ‘more than 20 years’ of experience in animal husbandry (*n* = 116, 48.5%). This was followed by ‘11 to 20 years’ (*n* = 45, 18.8%) and ‘5 to 10 years’ (*n* = 35, 14.6%). Lastly, 17 participants stated that they had ‘less than 5 years’ of experience (*n* = 17, 7.1%). Twenty-six participants did not answer this question (*n* = 26, 10.9%). Use of ‘conventional husbandry with outdoor access’ was the most common type of production system (*n* = 125, 52.3%), followed by ‘conventional husbandry’ (*n* = 92, 38.5%), ‘conventional husbandry with outdoor climate stimulus’ (*n* = 48, 20.1%) and lastly ‘biological/ecological animal husbandry’ (*n* = 43, 17.6%). The majority of farms kept their animals in only one type of animal production system (*n* = 182, 76.2%). The most common combination of production systems was ‘conventional husbandry’ and ‘conventional husbandry with outdoor access’ (*n* = 16, 6.7%), followed by the addition of a ‘conventional husbandry with outdoor climate stimulus’ system to the former combination on 15 farms (6.3%). Two participants did not answer the question concerning their production system type (*n* = 2, 0.8%). Chicken (*n* = 141, 59.0%), cattle (*n* = 106, 44.4%) and swine (*n* = 70, 29.3%) were the most prevalent species kept on the farms. More than half of the farms kept more than one animal species (*n* = 133, 55.6%), with a median of 2 (MAD = 1) animal species per farm. The most prevalent combinations were chicken and cattle (*n* = 52, 21.8%), chicken and swine (*n* = 29, 12.1%) and swine and cattle (*n* = 24, 10.0%) (Figure 3). Three farmers did not state which animal species were being kept (*n* = 3, 1.3%).

More than two thirds of all cattle holdings were categorized as beef cattle (*n* = 72, 67.9%), with 16 (15.1%) farms simultaneously keeping milk cattle and 49 (46.2%) keeping at least one other animal species on the farm. The majority of chicken holdings kept layer chicken (*n* = 121, 85.8%). Twenty farms also kept broiler chicken (*n* = 20, 14.2%) and 79 (56.0%) some kind of other animal species. Ninety percent of all farms stating to keep pigs categorized their animals as fattening pigs (*n* = 63, 90.0%). Half of them only kept pigs for fattening (*n* = 35, 50.0%), while the rest kept pigs of earlier stages of the production chain. Over half of the farms keeping pigs kept also another animal species (*n* = 37, 52.9%).

### 3.2. State of Biosecurity

**Figure 4:**
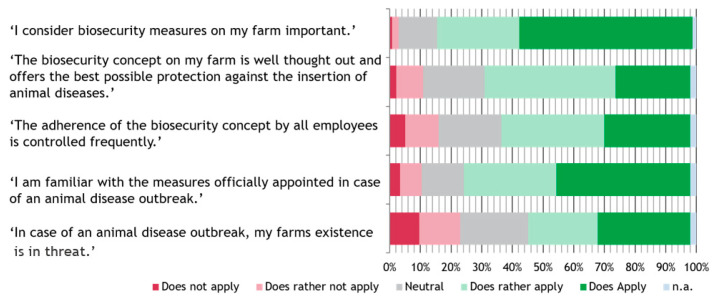
Likert scale for statements concerning the importance of biosecurity on the farm as stated by the participants.

All five statements about the importance of biosecurity on the farm were categorized as ‘rather applies’ or ‘applies’ by the majority of farmers. The strongest agreement was found for the statement ‘Importance’ (*n* = 199, 83.3%), followed by ‘Official measures’ (*n* = 176, 73.6%). This was followed by the statements ‘Concept’ (*n* = 160, 66.9%), ‘Adherence’ (*n* = 147, 61.5%) and ‘Threat’ (*n* = 126, 52.7%). Three participants did not rate any of the statements (*n* = 3, 1.3%) and one farmer each did not evaluate one, three and four answers, respectively (*n* = 1, 0.4%) (Figure 4).

**Figure 5:**
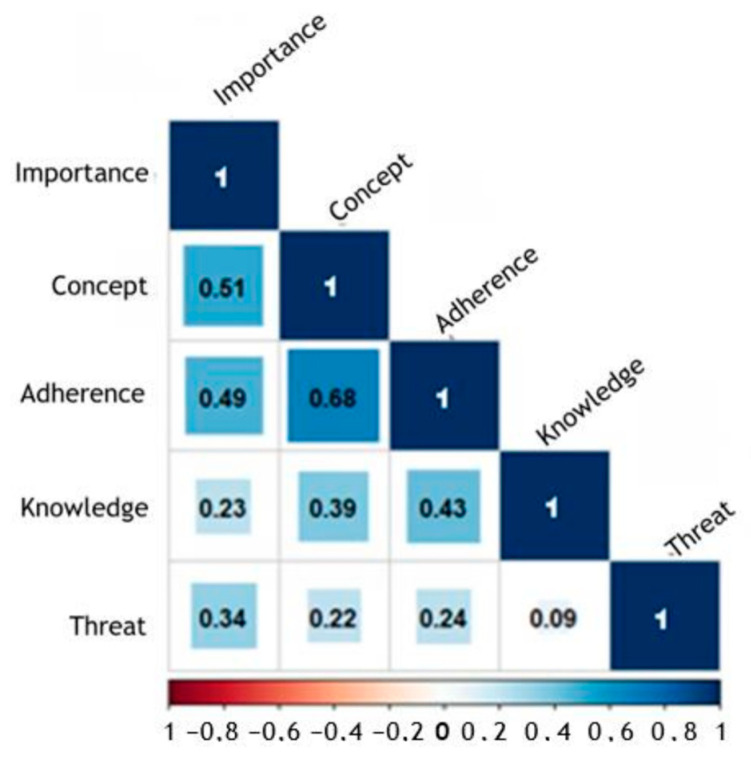
Spearman’s rank correlation coefficient (ρ) between answers given to statements about biosecurity in a Likert-scale format.

Two-hundred-and-thirty-three datasets (*n* = 233, 97.5%) were included in the Spearman’s ρ calculation. Spearman’s rank correlation showed significant correlation between each statement combination. However, a strong positive association was only found for the statement combinations ‘Adherence’ and ‘Concept’, with an ρ of 0.68, and ‘Importance‘ and ‘Concept‘, with an ρ of 0.51 (Figure 5).

Overall, the majority (*n* = 203, 84.5%) of farmers stated that there was a concept of biosecurity on their farm. The farmers received the information for their conception and development of biosecurity, in the largest percentage, from their veterinarian (*n* = 151, 63.2%). In addition, information was gathered from other farmers (*n* = 78, 32.6%), the animal health service (*n* = 73, 30.5%) and the chamber of agriculture (*n* = 62, 25.9%). Farmers consulted a median of two (MAD = 1) sources for the conception of the biosecurity concept. One farmer did not respond to the question (*n* = 1, 0.4%). Nearly two thirds of farmers (*n* = 152, 63.6%) stated that they evaluated their concept of biosecurity with the help of their veterinarian (*n* = 71, 29.7%), an online-tool (*n* = 47, 19.7%) or an institution (*n* = 34, 14.2%). Seven farmers did not answer this question (*n* = 7, 2.9%).

### 3.3. Assessment of Hazardous Situation

**Figure 6:**
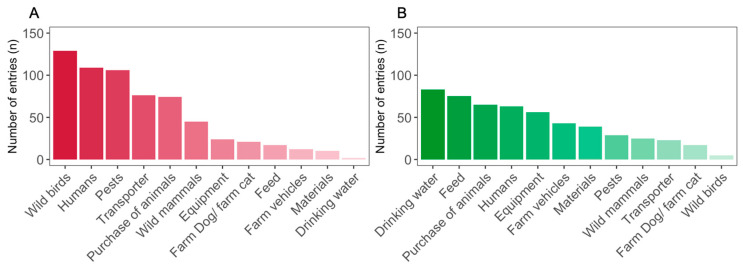
Pareto charts of the possible introduction routes for a disease into the farms (**A**) as well as the introduction routes perceived to have a high protection status (**B**). Number of entries are represented as bars.

The highest risk for an introduction of an animal disease into the farm was seen in the introduction routes ‘wild birds’ (*n* = 129, 54.0%), ‘humans’ (*n* = 109, 45.6%) and ‘pests’ (*n* = 106, 44.4%). Furthermore, the introduction routes ‘transporter’ (*n* = 76, 31.8%) and ‘purchase of new animals’ (*n* = 74, 31.0%) were selected by approximately one third of farmers. With three introduction routes being the maximum allowed in the questionnaire, farmers selected a medium of 3 (MAD = 0) answers, resulting in 625 introduction routes being selected all together. Sixteen farmers did not select any of the given introduction routes (*n* = 16, 6.7%) (Figure 6A).

The introduction routes that were described as best protected by the existing biosecurity measures were ‘drinking water’ (*n* = 83, 34.7%), ‘feed’ (*n* = 75, 31.4%), ‘purchase of new animals’ (*n* = 65, 27.2%) and ‘humans’ (*n* = 63, 26.4%). Overall, the introduction routes were selected 523 times, with the five most frequently selected introduction routes making up two thirds of all answers (*n* = 342, 65.4%). Farmers selected a median of three (MAD = 0) answers, and 57 farmers did not choose any introduction routes (*n* = 57, 23.9%) (Figure 6B).

**Figure 7:**
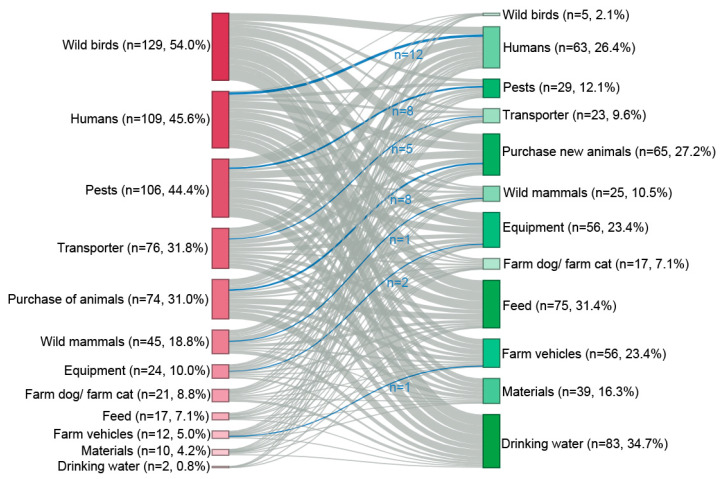
Risk factors (red), as well as introduction routes that were perceived as well protected (green) by the participants. Gray lines indicate their mutual selection by one participant, with the blue lines highlighting the selection of one introduction route in both categories by one participant. The color gradients of red and green change with increasing percentages (dark red: highest risk, dark green: best protected).

In most cases, the introduction routes that were labeled as highest risk were not marked as best protected by the same farmer. This was only the case for the introduction route ‘humans’ for twelve farmers (*n* = 12, 5.0%), ‘pests’ as well as ‘purchase of new animals’ (*n* = 8, 3.3%), ‘transporters’ (*n* = 5, 2.1%), ‘equipment’ (*n* = 2, 0.8%) and ‘wild mammals’ as well as ‘farm vehicles’ (*n* = 1, 0.4%) (Figure 7).

### 3.4. Options for Improvement

Farmers chose a median of two (MAD = 1) answers of the given nine options on possible offers for improved biosecurity on their farm. More than half of the farmers wanted regular information on the current state of animal disease outbreaks (*n* = 139, 58.2%) and short fact sheets (*n* = 131, 54.8%), as well as a mobile app (*n* = 81, 33.9%). Twenty-one farmers did not see a need for such offers (*n* = 21, 8.8%) and three did not answer the question (*n* = 3, 1.3%).

Twenty-seven participants filed out the open text field (*n* = 27, 11.3%). The information was classified into 13 themes (Supplementary S2). The majority of statements (*n* = 20, 74.1%) contained information that was assigned to a single theme. In addition, the information of six statements (*n* = 5, 18.5%) and two statements (*n* = 2, 7.4%) was assigned to two and three themes, respectively. Most statements discussed a general uncertainty about biosecurity (*n =* 6, 2.5%), focusing on the lack of absolute safety and the need for reliable instructions. The personal responsibility of the farmer was mentioned (*n =* 4, 1.7%) by highlighting the fact that everyone should start with themselves. Furthermore, the feasibility of biosecurity measures (*n =* 3, 1.3%) was discussed by mentioning structural and financial impediments. Some statements highlighted free-range holdings (*n* = 3, 1.3%) as well as hobby husbandry (*n =* 1, 0.4%) as the main risk source for animal disease outbreaks affecting larger, commercial farms in their area. In contrast, two statements mentioned the good information about biosecurity provided from the mobile stable association (*n* = 2, 0.8%). The role of veterinary officer was described (*n* = 4, 1.6%) as both helpful and difficult. In addition, two participants (*n* = 2, 0.8%) mentioned the psychological stress that farmers are exposed to as a result of an animal disease outbreak in the form of self-blame. Two farmers mentioned that there was unauthorized entry happening on their farm (*n* = 2, 0.8%). One farmer each highlighted the importance of biosecurity respective discrepancies in the family (*n =* 1, 0.4%). Four statements added further information about their farm (*n =* 4, 1.7%) and three criticized the design of the questionnaire (*n =* 3, 1.3%). Codes used in the generation of themes can be found in Supplementary S2.

## 4. Discussion

The implementation of daily management practices is essential for functioning biosecurity on a farm level. It relies on the attitude and the risk awareness of the people on site. Therefore, it is important that farmers actively engage with the topic of biosecurity. This fact is also reflected in the current European legislation. In the Animal Health Law, a direct focus is put on the responsibility of the farmer in terms of biosecurity. It states that the operators of farms are responsible for ‘minimizing the risk of the spread of diseases’. The large majority of farmers in the present study declared that they had a concept of biosecurity on their farm and thereby implied that they were actively taking charge on this topic. Furthermore, nearly two-thirds of farmers reevaluated their biosecurity concept with the help of an external institution, indicating a strong willingness to implement the best available biosecurity measures on their farm. In both the creation and the evaluation of their biosecurity plan, the consultation of a farm’s veterinarian was by far the most prevalent form of external input. The role of the veterinarian in terms of biosecurity has been shown before [36,37,38]. In a series of interviews with German pig holders on biosecurity in the context of ASF, a similar strong focus on trust in regards to their respective farm veterinarian was found, thus highlighting the practicability of their advice [39]. Veterinarians seem to be aware of their influence on farmers, as the majority state that they advise farmers on on-farm biosecurity [40,41,42,43,44]. However, studies suggest veterinarians do encounter problems in communicating relevant information to farmers [45,46]. Therefore, veterinarians should be educated during their studies, as well as later on through information campaigns, about biosecurity on the farm level and effective strategies on how to communicate with farmers [42,47]. In addition, veterinarians working in the field should be actively involved in the evaluation of mandatory biosecurity measures.

The attitude of farmers towards biosecurity was further explored by five statements about biosecurity. As the statement about the importance of biosecurity found a nearly one-hundred percent agreement, farmers seemed to recognize the importance of the topic. The strong correlation between this statement and the statement about the existence of a well thought-through concept of biosecurity seems to be reasonable, since the importance of a topic directly impacts the motivation to act on it. In addition, a strong correlation was found between the latter statement and the control of adherence to the biosecurity plan. The carrying out of daily practices according to an existing biosecurity concept was found to be, in general, poor as well as error-prone in execution [48,49]. Therefore, the active control of adherence to the concept by all people working on a farm seems to be essential for effective biosecurity on the farm. Since the majority of farmers agreed to this statement, this shows that participants in the present study were not only aware of the importance of biosecurity and a defined concept, but also about the need for active control of the implementation on their farm. In contrast, the weakest correlation was found between farmers that stated that they had a good knowledge of official appointed measures in the case of an animal disease outbreak and farmers that thought their farm’s existence would be under threat in such a scenario. Although no negative correlation was found, probably due to the small percentage of participants disagreeing to the statement ‘Knowledge’, this hints at a lack of knowledge in this area, which should be explored in further surveys. An outbreak of an animal disease listed in the European Animal health law requires the application of certain measures to contain the outbreak, such as culling of the whole stock [50]. Even though the loss is compensated for by the responsible authorities, this reparation only includes the animal’s market value and no other costs arising during an outbreak on the farm [51]. Recently, in parts of the federal state Lower Saxony, this compensation was partially reduced when breaches of the biosecurity regulations were identified on the affected farm by the authorities [52]. Therefore, it seems reasonable that over half of the farmers participating did fear for their farm’s existence in case of an outbreak happening on their farm.

Introduction routes of animal diseases to the farms perceived by farmers as dangerous showed a uniform picture. This was shown by the fact that the four most frequently named introduction routes made up the majority of answers given and that nearly all farmers answered this question. The ranking of farmers in the present study showed similarities with the results of expert panels that were asked to weigh subcategories of biosecurity against each other in terms of importance [53,54]. Since the human aspect, animal trade and pest control were, among others, the factors with the greatest weight assigned, it seems that farmers perceived the risk posed by the different introduction routes similarly to experts in the field. This further supports the already discussed engagement of farmers in the topic of biosecurity. In contrast, the question about introduction routes that were well protected by existing measures was not answered by a quarter of farmers, and there was much more variation in this question than in the previous one. Both the diversity of selected introduction routes as well as the large group of farmers not selecting any introduction route hints at a general insecurity about the effectiveness of biosecurity. Rating the introduction routes as best protected implies a certain knowledge about the effectiveness of existing measures. Even when looking into the scientific literature, it is hard to quantify the effectiveness of biosecurity measures, which is a situation that is acknowledged by veterinary experts [55,56]. Due to the lack of quantitative data, existing scores designed to rate the biosecurity of a farm as a whole rely on expert opinions for their weights of the importance of different measures [53,54,57,58]. In that context, the discrepancy between the selection of dangerous introduction routes and the selection of good protected introduction routes was obvious, both at a general level and on a single farmer’s choice level. Additionally, when looking at a single farmer’s choice, introduction routes considered dangerous to the farm were only considered rarely as well-protected introduction routes. This further emphasizes the problem that even though farmers identified introduction routes of concern in their biosecurity concept, they apparently lacked knowledge about possibilities for improvement. The survey did not investigate the underlying causes of farmers’ uncertainties regarding the effectiveness of biosecurity measures. Due to the ongoing dynamics of the animal disease situation in Germany, farmers were increasingly being sensitized to the significance and importance of biosecurity via information events, veterinary consultations and political influences. One reason for the uncertainty with regard to effectiveness could be that, although training has emphasized the importance of biosecurity, the selection, prioritization and implementation of targeted biosecurity measures has been neglected. Furthermore, biosecurity measures are very farm-specific and dependent on the actual implementation, which makes it difficult to compare the measures applied across farms. This could make it difficult for farmers to assess whether measures are relevant for their farm or not, due to their type of production. In addition, the aim of biosecurity is not to introduce an animal disease into the farm; thus, as long as the farm has a high health status, the success of the biosecurity measures and their effectiveness are only indirectly visible and therefore difficult for farmers to quantify. Furthermore, it may be difficult for farmers to identify whether the health status of their herd is due to the biosecurity measures implemented or whether the farm is currently not exposed to any potential pathogens.

Lastly, open text field question entries hinted at further problems that concern farmers in the context of biosecurity. Foremost, the mentioned uncertainty of biosecurity in combination with the feasibility of biosecurity measures on farms further highlight the need for both further research on the topic as well as clear communication from the authorities to farmers. This is in line with the conclusions found in the aforementioned series of interviews with German pig holders [39] and a similar study with UK broiler farm workers [59]. Furthermore, the open free-text answers suggested tensions between conventional farmers and small-holder farms concerning biosecurity on free-range holdings. Since the already described compensation in case of a listed animal disease outbreak is only paid to the affected farm and not to farms in the region affected by further measures, such as restriction zones with a ban on animal transportation, farms are directly impacted by the situations of their neighboring farms. A similar conflict seems to be happening in the UK [60] and should be further explored in the German context. The resulting peer pressure also relates to the psychological impact on farmers that was mentioned in the free-text section. In that context, it should not be forgotten that farmers are mostly people building their and their family’s existence around the existence and profitability of their farm [61]. Keeping the high suicide rate of farmers in mind [62], authorities should aim for clear communication of reasonable measures and see farmers as partners in the common goal of achieving the best biosecurity possible.

A limitation of this study might be that the participating group of farmers showed some deviations from the overall group of farmers in Germany, as can be estimated from the available data from official state sources. However, the location of participating farms complied with farms in Germany on the level of the three main species kept. Furthermore, as the average age of farmers in Germany was estimated to be 53 in 2013 [63] and recent data indicate that 85% of farm managers in Germany are above the age of 40 [64], the overall high experience of animal husbandry in the participating group seems reasonable. In contrast to these two general information criteria, animal species kept did not represent the situation on German farms. The most prevalent animal species kept on German farms for food production are cattle (64%) followed by chicken (30%) and pigs (19%) [65]. In the present study, those three species were also by far the most frequently species kept. However, chicken and pigs were found in a higher percentage than expected. This might be due to the two most threatening animal diseases circulating in Germany right now, being ASF [66] and HPAI [67], which target these particular species and lead to high economic losses. Farmers keeping those species might be, in general, more engaged in the topic of biosecurity, raising their likelihood of participating in a survey about the topic [68]. Similar, the percentage of farms with organic husbandry exceeded the estimated 10% in Germany [69]. The percentage of conventional husbandry with outdoor contact is not systematically collected for pigs [70] and cattle in Germany. For layer chicken, which is considered to be the sector of animal production with the highest percentage of husbandries with outdoor contact, 21% of all farms hold layer chicken in a free-range system [71], which is considerably less than the more than half of our participants stated to have a conventional husbandry with outdoor access on their farm. Both organic and conventional husbandries with outdoor access have a higher risk of contracting a disease into their animal stock since they are more exposed to the outdoor world and biosecurity measures are harder to implement [72,73,74]. Therefore, as mentioned above in the context of the over-representation of pigs and chicken, an increased engagement with the topic due to the higher risk in these holdings with outdoor contact might have led to a higher participation rate [68].

Overall, the participating subgroup of German farmers keeping animals for food production constituted a regionally representative cross-section with a focus on farmers keeping pigs and/or chickens in husbandries with contact to the outdoor world. Since this group of farmers is actually the group that is most challenged by the current animal disease situation in Germany, the collected information is very valuable in order to develop targeted strategies to help these farmers improve the biosecurity of their husbandry.

## 5. Conclusions

The high percentage of farmers actively engaging in the topic of biosecurity concerning animal husbandry indicates a general willingness to implement management practices on a daily basis. However, the underlying discrepancy of the perception of dangerous as well as well-protected introduction routes for pathogens hints at a potential lack of knowledge of the effectiveness of biosecurity measures. This displays a need for further research on this effectiveness and clear recommendations for farmers, which were also asked for by the participants.

## Figures and Tables

**Table 1:** Statements about biosecurity assessed according to a five-point Likert scale.

Statement	Abbreviation
‘I consider biosecurity measures on my farm important.’	‘Importance’
‘The biosecurity concept on my farm is well thought out and offers the best possible protection against the introduction of animal diseases.’	‘Concept’
‘The adherence of the biosecurity concept by all employees is controlled frequently.’	‘Adherence’
‘I am familiar with the measures officially applied in case of an animal disease outbreak.’	‘Official measures’
‘In case of an animal disease outbreak, my farm’s existence is under threat.’	‘Threat’

## Data Availability

The data presented in this study are available upon reasonable request from the authors.

## Supplementary Materials

The following supporting information can be downloaded at: https://www.mdpi.com/article/10.3390/ani14071102/s1, Supplementary S1: Survey questionnaire, Supplementary S2: Themes and corresponding codes from the open text field question about further thoughts on biosecurity.
