# Supplementary material for: Risk Awareness and Attitude of German Farmers towards Biosecurity Measures
Source: Animals (Basel). 2024 Apr 4;14(7):1102. doi: 10.3390/ani14071102 (PMC11010927; doi:10.3390/ani14071102)
Supplement: Supplementary file 1 [file animals-14-01102-s001.zip › Supplementary/S1_survey_questionnaire.pdf]

Starting page

ÖTiKlis

0% completed

Survey about biosecurity

The ÖTiKlis project is a joint research project of the Friedrich-Loeffler-Institute and the Weihenstephan-Triesdorf University of Applied Sciences. The aim is to investigate the compatibility of ecology, animal health and animal disease control. For more information on the project, please visit the project page.

In this anonymous survey we ask you to share your experience as well as your opinion on biosecurity!
You will be asked a total of ten short questions. The estimated completion time is five to ten minutes.

The results of the survey will be used to create recommendations for action and measures to improve biosecurity on livestock farms. By participating in the survey, you are making an important contribution to this effort. Thank you for your assistance.

In addition to the survey presented here, we are looking for livestock farms to participate in a biosecurity survey. Information on how to participate can be found further down the page. We look forward to hearing from you!

Start survey!

Farms wanted to participate in the study!

Within the framework of the ÖTiKlis project, a comparative study on the topic of biosecurity in conventional and organic animal husbandry is planned.

If you are interested in participating, please contact us!

We are currently looking for pig and poultry farms of all types to participate. A study with cattle farms is [illegible]

+

Contact details

ÖTiKlis, Friedrich-Loeffler-Institut, Bundesforschungsinstitut für Tiergesundheit – 2023

Page 1

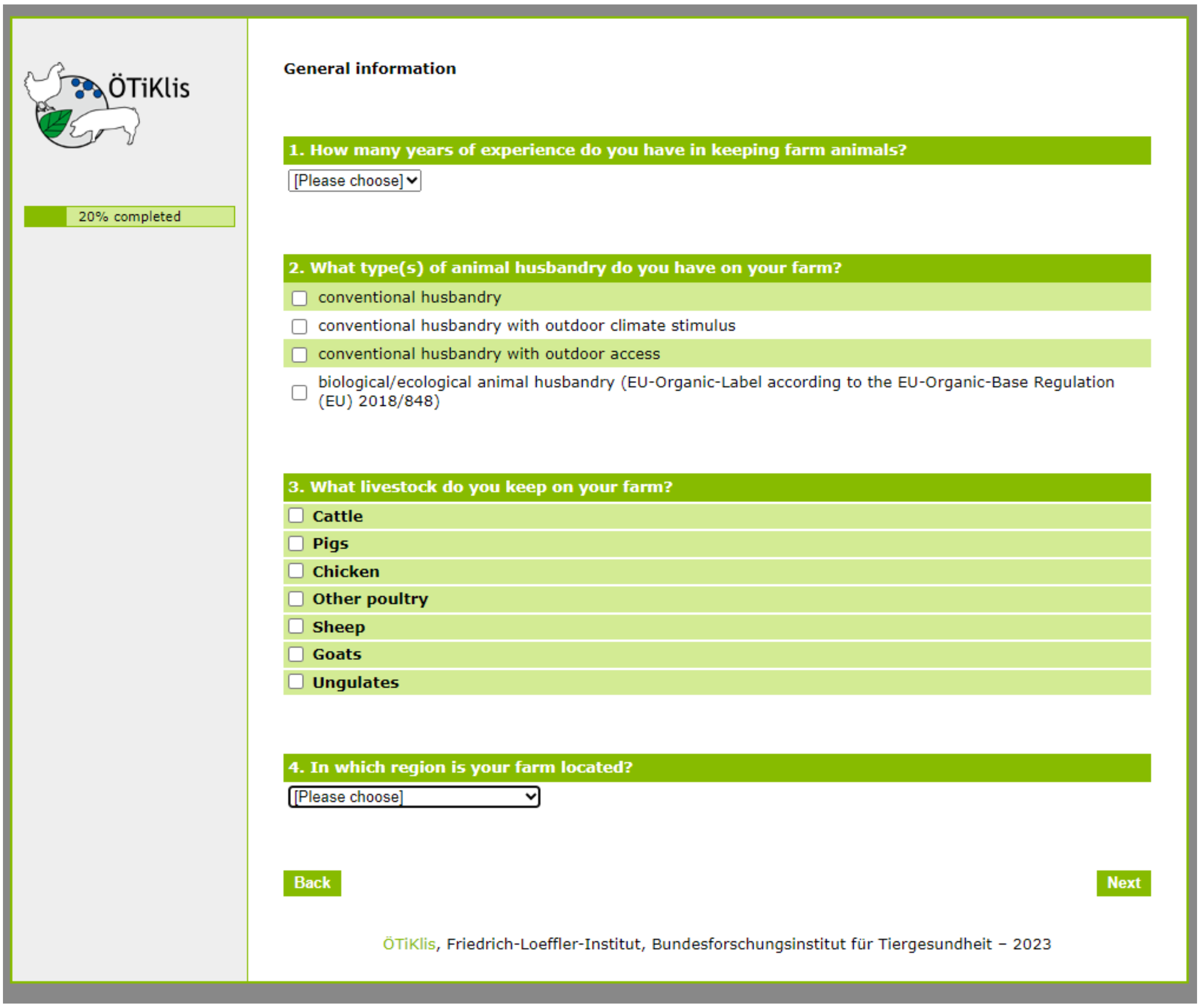

Answer options

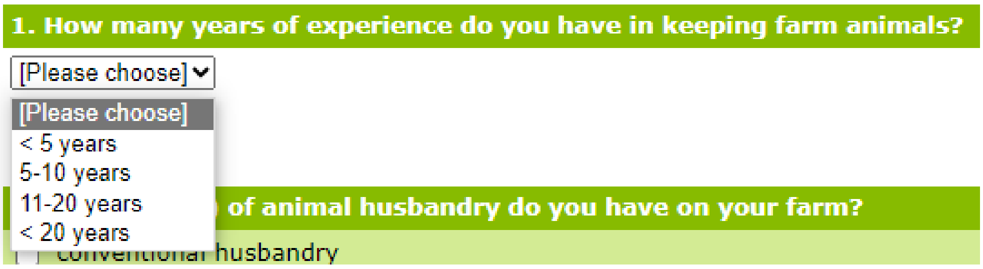

Example:

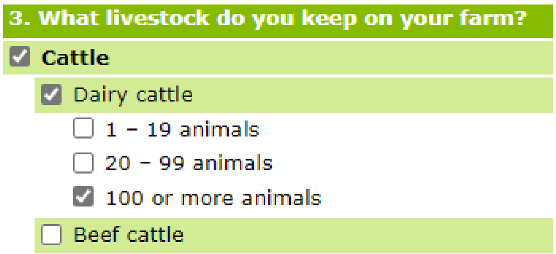

Scheme:

Category	Subcategory	Number of animals
Cattle	Dairy cattle	1 -19
		20 - 99
		≥ 100
	Beef cattle	1 -19
		20 - 99
		≥ 100
Pigs	Sows	1 -99
		100 - 999
		≥ 1.000
	Suckling piglets	1 -99
		100 - 999
		≥ 1.000
	Fattening piglets	1 -99
		100 - 999
		≥ 1.000
	Young pigs	1 -99
		100 - 999
		≥ 1.000
	Fattening pigs	1 -99
		100 - 999
		≥ 1.000
	Boar	1 -9
		≥ 10

Category	Subcategory	Number of animals
Chicken	Broiler	1 -99
		100 - 999
		≥ 1.000
	Layer	1 -99
		100 - 999
		≥ 1.000
	Dual-purpose	1 -99
		100 - 999
		≥ 1.000
Other poultry	Turkeys	1 -99
		100 - 999
		≥ 1.000
	Geese	1 -99
		100 - 999
		≥ 1.000
	Ducks	1 -99
		100 - 999
		≥ 1.000
Sheep		1 -19
		20 - 99
		≥ 100
Goats		1 -19
		20 - 99
		≥ 100
Ungulates	Horse	
	Donkey	
	Mule	

4. In which region is your farm located?

[Please choose]

biological/ecological animal husbandry (EU-Orga

- [Please choose]
- Baden-Württemberg
- Bavaria
- Berlin
- Brandenburg
- Bremen
- Hamburg
- Hesse
- Mecklenburg-Western Pomerania
- Lower Saxony
- North Rhine-Westphalia
- Rhineland-Palatinate
- Saarland
- Saxony-Anhalt
- Saxony
- Schleswig-Holstein
- Thuringia
- ——————————————
- Austria
- Switzerland

o on your farm?

m located?

[Please choose]

Page 2

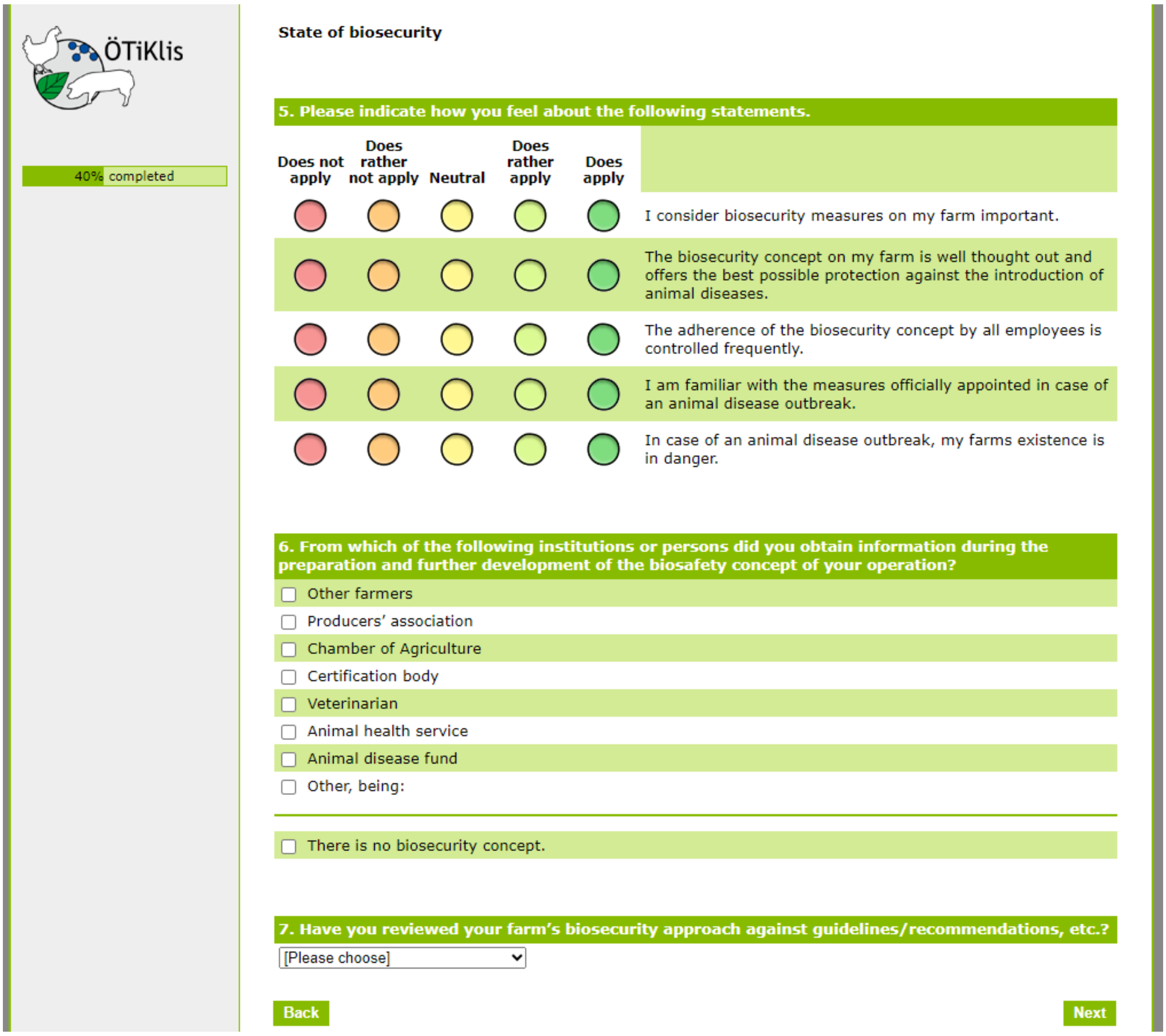

ÖTiKlis

40% completed

State of biosecurity

5. Please indicate how you feel about the following statements.

Does not apply	Does rather not apply	Neutral	Does rather apply	Does apply	
○	○	○	○	○	I consider biosecurity measures on my farm important.
○	○	○	○	○	The biosecurity concept on my farm is well thought out and offers the best possible protection against the introduction of animal diseases.
○	○	○	○	○	The adherence of the biosecurity concept by all employees is controlled frequently.
○	○	○	○	○	I am familiar with the measures officially appointed in case of an animal disease outbreak.
○	○	○	○	○	In case of an animal disease outbreak, my farms existence is in danger.

6. From which of the following institutions or persons did you obtain information during the preparation and further development of the biosafety concept of your operation?

- ☐ Other farmers
- ☐ Producers' association
- ☐ Chamber of Agriculture
- ☐ Certification body
- ☐ Veterinarian
- ☐ Animal health service
- ☐ Animal disease fund
- ☐ Other, being:

- ☐ There is no biosecurity concept.

7. Have you reviewed your farm's biosecurity approach against guidelines/recommendations, etc.?

[Please choose]

Back Next

Answer options

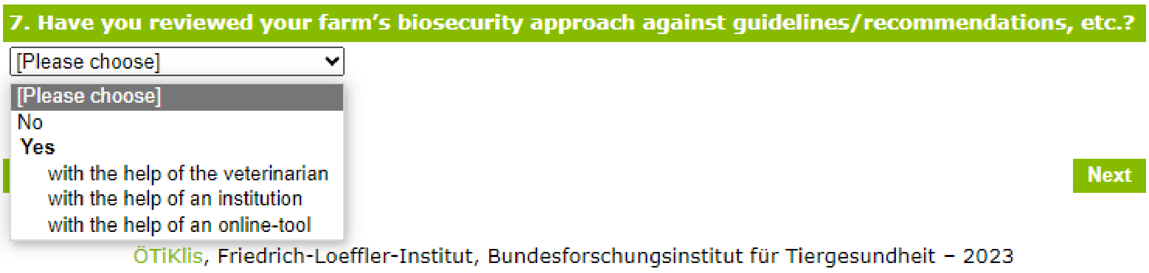

7. Have you reviewed your farm's biosecurity approach against guidelines/recommendations, etc.?

[Please choose]

[Please choose]
No
Yes
with the help of the veterinarian
with the help of an institution
with the help of an online-tool

Next

ÖTiKlis, Friedrich-Loeffler-Institut, Bundesforschungsinstitut für Tiergesundheit – 2023

Page 3

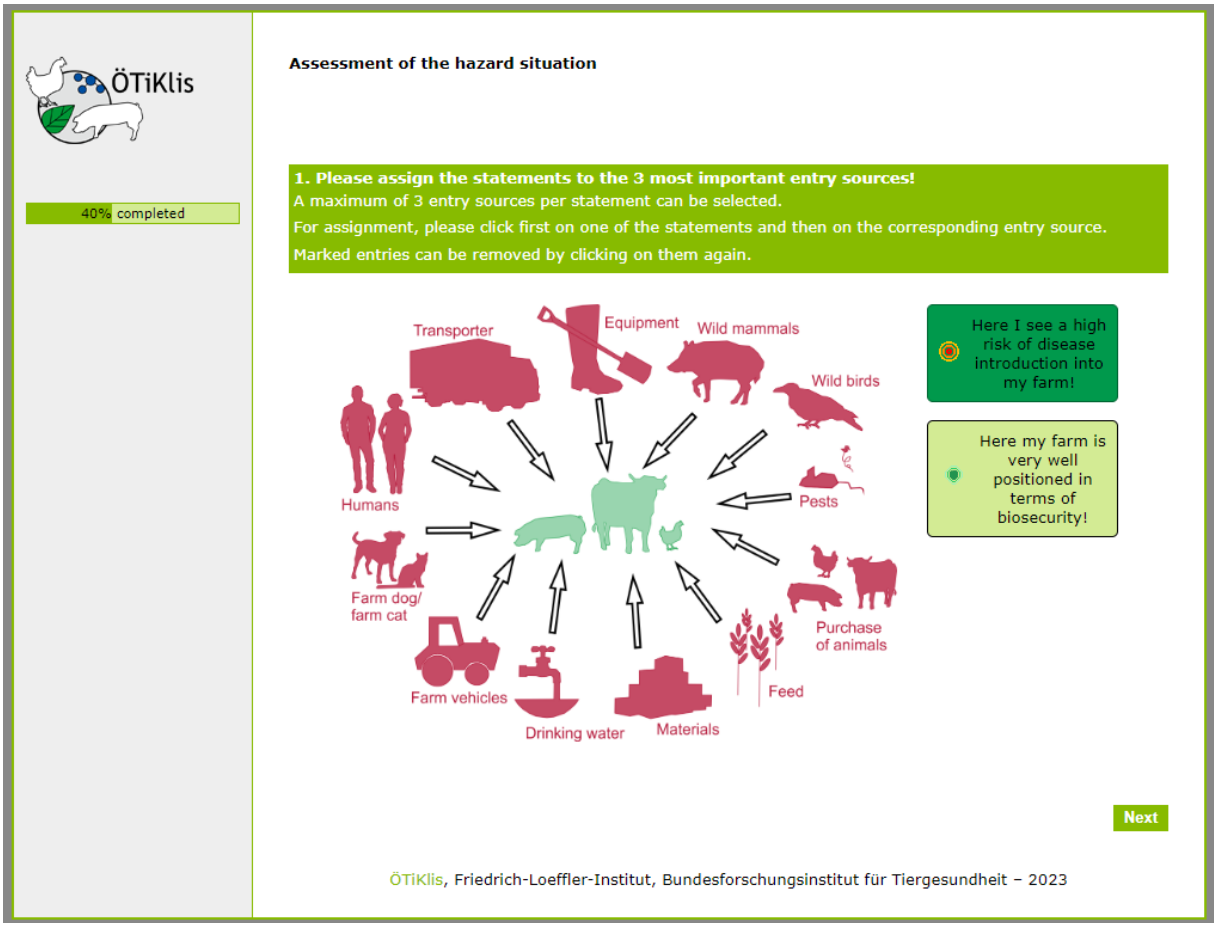

Filled out example for page 3

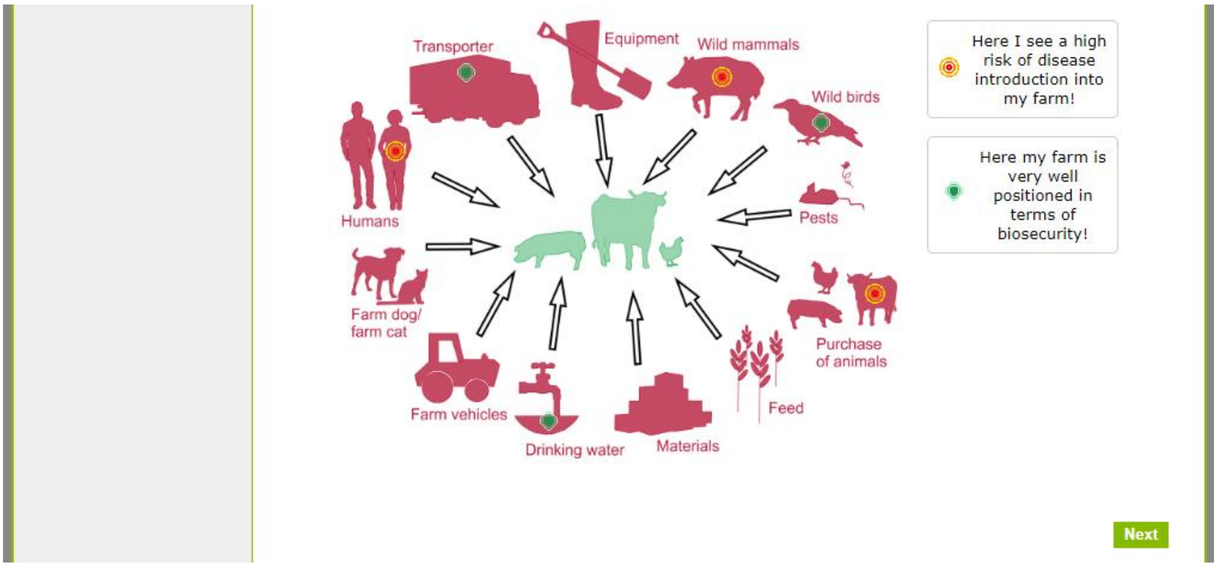

Page 4

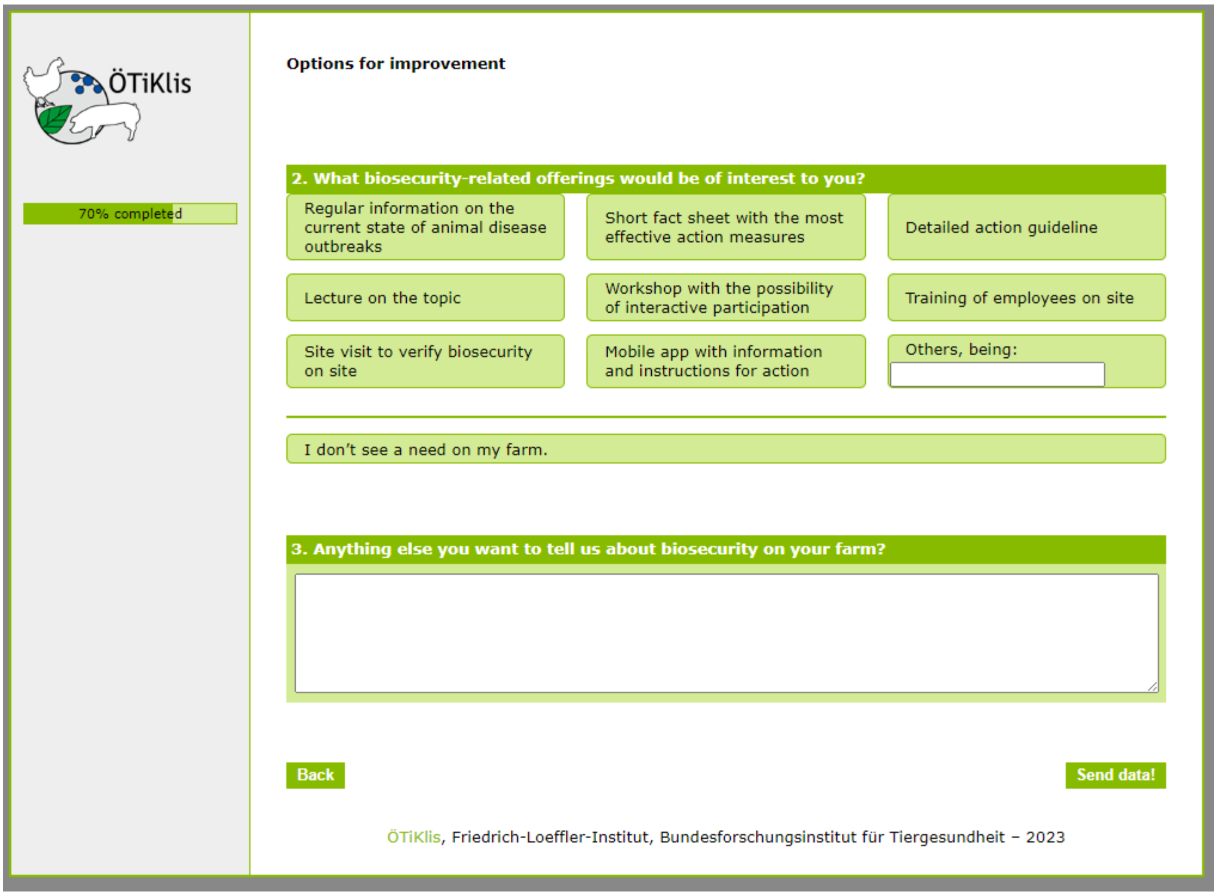

ÖTiKlis

70% completed

Options for improvement

2. What biosecurity-related offerings would be of interest to you?

Regular information on the current state of animal disease outbreaks	Short fact sheet with the most effective action measures	Detailed action guideline
Lecture on the topic	Workshop with the possibility of interactive participation	Training of employees on site
Site visit to verify biosecurity on site	Mobile app with information and instructions for action	Others, being:

I don't see a need on my farm.

3. Anything else you want to tell us about biosecurity on your farm?

Back

Send data!

ÖTiKlis, Friedrich-Loeffler-Institut, Bundesforschungsinstitut für Tiergesundheit – 2023

Last page

Thank you very much for your participation!

Your answers will help us to better assess the attitudes and experiences of German agriculture on the topic of biosecurity.

The results of the survey will be published on the ÖTiKlis project page after the end of the survey period.

We would like to thank you very much for your assistance.

ÖTiKlis, Friedrich-Loeffler-Institut, Bundesforschungsinstitut für Tiergesundheit – 2023
