# Supplementary material for: Risk Awareness and Attitude of German Farmers towards Biosecurity Measures
Source: Animals (Basel). 2024 Apr 4;14(7):1102. doi: 10.3390/ani14071102 (PMC11010927; doi:10.3390/ani14071102)
Supplement: Supplementary file 1 [file animals-14-01102-s001.zip › Supplementary/S2_Themes and corresponding codes from the open text field question about further thoughts on biosecurity.pdf]

Themes and corresponding codes from the open text field question about further thoughts on biosecurity.

Theme	Code(s)
Uncertainty about biosecurity	Need for valid information
	Reliable instructions in advance
	Need for reasonable ASF control
	Everything possible is implemented, but still uncertainties (e.g. wild-birds)
	There will always be unpredictability
	There is no absolute safety
Personal responsibility	Concepts on paper are of no use on their own
	Consistently implementation in practice
	Starting with oneself; not pointing the finger at others first
Further information	Grazing with mobile shelter
	Home slaughtering
	No problems during many decades
	The farm is now shut down
Free-range holdings	The biggest thread is the neighboring free-range holding

	In free-range holdings the biggest thread are wild-birds
	Biosecurity is hard to implement in free-range holdings
Design of the questionnaire	Setting options should be reconsidered
	Additional category for extensive beef cattle breed
	Last statement of the Likert-Scale is unclear about location of disease outbreak
Feasibility of biosecurity measures	Evolved structures hinder implementation of measures
	Compromise between different requirements (time-workload, costs-yield)
	Concepts are not implemented
Veterinary officer	Some veterinary officers don't know what to do
	Local veterinary officers are doing a good job
	Cooperation and active exchange with veterinary officers
	Not enough time for inspections
Unauthorized entry	Unauthorized access to the premises despite the fact that the premises are closed
	Unauthorized intrusion of NGOs
Psychological impact	Self-reproach in the event of an outbreak on one's farm
	Disease protection is self-protection

	Open-communication is needed
	Some prefer to remain-silent
Mobile stable association	Mobile stable association supplements very good information material
	Mobile stable association is good at support
Hobby husbandry	Hobby holders display no personal responsibility
Discrepancies in the family	Other family members do not see the relevance of biosecurity
Importance of biosecurity	Very important for every farm
